# Statistics of the Profession in Paris

**Published:** 1866-06

**Authors:** 


THE PROFESSION IN PARIS.—There are in the French capital 1848 doctors of medicine 375 medical men of the second grade, and 740 midwives. Among these, 340 are members of the Legion of Honor, 94 are officers of the same legion, 26 commanders, 1 grand officer, and 1 grand cross. There is about one medical man for 800 inhabitants, reckoning the population at 1,800,000, and taking the whole of the department of the Seine, which includes 2223 professional men.—*London Lancet.*

